# Risk Factors Associated With the Development of Nephropathy 10 Years After Diagnosis in Taiwanese Children With Juvenile-Onset Type 1 Diabetes—A Cohort Study From the CGJDES

**DOI:** 10.3389/fendo.2018.00429

**Published:** 2018-08-03

**Authors:** Ching-Chien Yang, Chia-Hung Lin, Nan-Kai Wang, Chi-Chun Lai, Fu-Sung Lo, The Chang Gung Juvenile Diabetes Eye Study Group, Tun-Lu Chen

**Affiliations:** ^1^Division of Pediatric Endocrinology and Genetics, Department of Pediatrics, Chang Gung Memorial Hospital, Linkou Medical Center, Taoyuan, Taiwan; ^2^Division of Endocrinology and Metabolism, Department of Internal Medicine, Chang Gung Memorial Hospital, Linkou Medical Center, Taoyuan, Taiwan; ^3^Department of Medicine, College of Medicine, Taoyuan, Taiwan; ^4^Department of Ophthalmology, Chang Gung Memorial Hospital, Linkou Medical Center, Taoyuan, Taiwan

**Keywords:** type 1 diabetes mellitus, diabetic nephropathy, adolescents, children, risk factors

## Abstract

**Objective:** To examine the risk factors for diabetic nephropathy (DN) 10 years after the diagnosis of juvenile-onset type 1 diabetes mellitus (T1DM) in a Taiwanese population.

**Research Design and Methods:** This retrospective, observational, longitudinal cohort study of 224 patients with T1DM for >10 years (mean duration 12.6 years) included participants from the Chang Gung Juvenile Diabetes Eye Study Group. The patients received a T1DM diagnosis before the age of 18 years and were treated at the pediatric endocrine department of Chang Gung Memorial Hospital in Taiwan. The epidemiological and laboratory data such as age, sex, duration of diabetes, self-reported smoking, blood pressure, lipid profiles, urinalysis, and glycated hemoglobin A1c (HbA1c) levels were collected from medical records retrospectively for investigating the relationship between the clinical parameters and the development of DN in T1DM.

**Results:** During follow-up, 44 of the 224 patients (19.6%) developed DN, of whom 61.4% were female. Cox proportional hazards model analysis indicated that the female (HR 3.40, 95% CI 1.66–6.96, *p* = 0.001), smoking (HR 3.60, 95% CI 1.28–10.10, *p* = 0.015), HbA1c level (HR 1.27, 95% CI 1.07–1.49, *p* = 0.005), diastolic blood pressure (HR 1.06, 95% CI 1.03–1.09, *p* < 0.001) were significantly correlated with DN after adjustment for multiple variables. The tight glucose control with multiple daily injections produced 49 % risk reduction (HR 0.51, 95% CI 0.26–0.98, *p* = 0.043).

**Conclusions:** The risk of DN in patients with juvenile-onset T1DM 10 years after the T1DM diagnosis was increased with female, smoking, high HbA1c, diastolic blood pressure levels and attenuated by intensive therapy.

## Introduction

The prevalence of type 1 diabetes mellitus (T1DM) has increased from 2 to 5% according to large epidemiological studies worldwide. Moreover, T1DM continues to account for >90% of cases of juvenile-onset diabetes (1, 2). In 2010, the prevalence of diagnosed diabetes in the youth population was estimated at 2.13/1,000 persons in the United States; this number may triple by 2050, with the highest increase occurring in minority racial and ethnic groups (3). Diabetic macro- and microvascular complications result in increased disability and substantial health care costs. The only modifiable predictor was glycemic control for the development and progression of microvascular complications in childhood onset type 1 diabetes (4).

Although studies have analyzed diabetic nephropathy (DN) in childhood T1DM, few studies have focused on Asian patients. The degree of albuminuria is not necessarily linked to disease progression in patients with T1DM-associated DN. Therefore, the factors responsible for DN must be clarified, particularly those that cause DN in juvenile-onset T1DM patients. The decline in renal function is predominantly linear for individual patient, but the slopes vary widely among patients. The lifetime risk of DN in T1DM patients varies among studies and ethnic groups (5–8). Recently, researchers indicated an effect of sex on hyperfiltration in patients with T1DM without complications (9). Female patients with T1DM and DN had higher renal efferent arteriolar resistance and attenuating protection against the development of renal complications in the hyperfiltration state than male patients. However, the prevalence DN was higher among male patients than among female patients in the Taiwan National Health Insurance Research Database (10). But none of them had focused on the juvenile-onset population and been lack of long-term follow-up data.

End stage renal disease (ESRD) develops many decades after T1DM onset due to renal decline (3, 11). The decline trajectory and underlying mechanisms are insufficiently understood as a result of large differences in the distribution of risk factors for T1DM among different populations and racial groups. Because the rate of ESRD is higher in Taiwan (12) than in other countries, identifying the risk factors for T1DM that have a large impact on children and adolescents is important. We have published the retinopathy result from the cohort of Chang Gung Juvenile Diabetes Eye Study (CGJES) (13) but found different risk factors in the subsequent analysis for nephropathy in children with type 1 diabetes in Taiwan. The objective of this study was to determine whether these risk factors were associated with DN 10 years after the diagnosis of T1DM in a contemporary cohort of patients with juvenile-onset T1DM in Taiwan.

## Materials and methods

### Diabetes shared care program

In Taiwan, the Diabetes Shared Care Program (DSCP) was established in 1996 with the support of the Bureau of Health Promotion of the Department of Health. Our institute joined the DSCP in 2005 (14). The program provides integrated patient care by a team of physicians, diabetes specialists, nurses, and dietitians. All patients receive individual instruction from certified diabetes specialists, nurses, and dietitians. Patients also undergo non-mydriatic fundus photography annually and are referred to ophthalmologists for further dilated fundus examination if any abnormality is observed.

### Subjects

This retrospective study used the cohort of the ongoing Chang Gung Juvenile Diabetes Eye Study, collecting comprehensive data from patients with juvenile-onset T1DM to identify genetic and environmental risk factors for diabetic complications (13). The diagnosis of T1DM was based on the American Diabetes Association (ADA) recommendations (15). Recruitment was randomly done without considering the underlying characteristics, and the inclusion criterion was T1DM diagnosed before the age of 18 years. Hence, the sampling or selection procedure was done without selection bias. This study was carried out in accordance with the recommendations of the Institutional Review Board of Chang Gung Memorial Hospital, Taiwan with written informed consent from all subjects. Written informed consent was obtained from all subjects and the parents of the participants under the age of 16 in accordance with the Declaration of Helsinki. The protocol was approved by the Institutional Review Board of Chang Gung Memorial Hospital, Taiwan (103-3203B).

In this study, 99% patients joined the DSCP and had regular outpatient visits every 1–3 months. During the scheduled annual appointment, the patients received a blood examination to monitor their hemograms, renal and liver functions, lipid profiles, and C-peptide and glycated hemoglobin A1c (HbA1c) levels. If the patients missed the appointment, they could be re-arranged on the nearby date to complete the exam. The patients' sexes, ages at onset, smoking statuses, body mass indices (BMIs), blood pressure (BP), and insulin treatment data at every visit were obtained from the medical records. No patient had taken any antidyslipidemic or antihypertensive agents in this study.

T1DM was diagnosed according to the clinical criteria recommended by the World Health Organization (16). Blood samples were examined at the central laboratory of Chang Gung Memorial Hospital. The HbA1c levels were measured through high-pressure liquid chromatography (Bio-Rad, Hemel Hempstead, U.K.). The urine albumin/creatinine ratio, serum cholesterol, low-density lipoprotein cholesterol (LDL-C), and triglyceride (TG) levels were measured using a biochemical analyzer (LABOSPECT 008, Hitachi High-Techonologies, Tokyo, Japan). C-peptide was measured by a solid-phase, two-site chemiluminescent immunometric assay (IMMULITE 2000 C-peptide assay, Siemens AG, Erlangen, Germany).

### Nephropathy

At the scheduled annual appointment, each patient provided the first morning urine sample for measurement of the ACR. The diagnosis of DN was considered if diabetic patients developing abnormal ACR defined by an ACR ≥30 mg/g (sustained, ≧2 consecutive measurements that were ≧4 weeks apart) in the absence of signs or symptoms of other primary causes of kidney damage according to ADA recommendations (15). The patients only manifested isolated proteinuria without red cell casts and had normal complement titers, negative rheumatoid factor and autoantibodies such as antinuclear antibodies (ANA) and anti-double-stranded DNA antibodies. Furthermore, the causes of kidney diseases such as glomerulonephritis caused by infections (e.g., hepatitis B and C) or autoimmune disease (e.g. systemic lupus erythematous) were excluded.

### Retinopathy

All patients underwent an annual ophthalmic evaluation by retinal specialists. The DR severity was classified according to the Early Treatment of Diabetic Retinopathy Study. In this study, DR was defined as any class of diagnosed retinopathy.

### The association between the clinical parameters and diabetic nephropathy

In this study, the epidemiological and biochemistry parameters, including age, sex, age at T1DM onset, duration of diabetes, self-reported smoking history (at least one cigarette in 1 month), arterial hypertension, lipid profile, glycemic control, and BMI, were analyzed with the aim of identifying the possible risk factors of DN. Furthermore, we also measured the ratio of total cholesterol (TC) to high-density lipoprotein cholesterol (HDL-C), which is a known indicator of cardiovascular risk (17). The choice of candidates for diabetic nephropathy was based on routinely available clinical parameters in our caring system. If the factors were not thoroughly collected from every subject, we did not include them for analysis. The HbA1c, BP and lipid profiles were assessed using the means for the entire study period. The BMI measurement, urinalysis, and ophthalmic evaluations were assessed using the most recent data. Other laboratory parameters from patients with DN collected before the onset of DN were analyzed.

### Statistical analysis

All results were expressed as the means ± standard deviation, and a *p* < 0.05 was considered significant. Continuous variables were assessed using the independent-sample *t*-test or the Mann–Whitney *U* test depending on the normality of distribution in each group. Categorical variables were assessed individually using the Chi-square test, and Fisher's exact test was performed for samples with expected values <5. The multiple comparisons were not considered at the current study design because these different factors were not similar or related and would not cause family-wise error.

Univariate Cox proportional hazards regression models were used to assess the associations between the following variables and the risk of DR: sex, age at onset of T1DM, mean HbA1c level, blood pressure, lipid profile (the total cholesterol, LDL-C and log-transformed TG levels), insulin dose and injection times. After the univariate analyses, a multivariable Cox proportional hazards model with backward model selection was used to identify risk factors for DN. The results are reported as probability values (p), hazard ratios (HRs), and 95% confidence intervals (CIs).

The Kaplan–Meier method was used for the survival analysis, and the log-rank test was performed to compare patients grouped according to the significant risk factors. Statistical analyses were conducted using the SPSS software (version 17.0; IBM, Armonk, NY, USA).

## Results

Overall, 224 patients (44.2 % female) met the inclusion criterion. The mean (SD) age at T1DM onset was 7.6 (3.9) years, and the mean duration (range) of diabetes was 12.6 (10–15) years. By the end of follow-up, 44 (19.6 %) of 224 patients developed DN. No subject was lost for follow-up. The clinical and biochemical characteristics of the patients with and without DN are listed in Table 1.

**Table 1:** Clinical and biochemical characteristics among patients with and without diabetic nephropathy.

	**All patients *n* = 224**	**With DN *n* = 44 (19.6)**	**Without DN *n* = 180 (80.4)**	***p***
Female sex	99 (44.2)	27 (61.4)	72 (40.0)	0.011^*^
Age at diabetes onset (years)	7.6 ± 3.9	7.9 ± 3.5	7.5 ± 4.0	0.517
<5	57 (25.4)	8 (18.2)	49 (27.2)	0.217
≥5	167 (74.6)	36 (81.8)	131 (72.8)	
Duration of diabetes (years)	12.6 (10–15)	13.1 (9.5–15.5)	12.4 (10.1–14.9)	0.322
Height (cm)	152.3± 14.9	153.5 ± 12.3	152.1 ± 15.5	0.510
Weight (kg)	47.6 ± 13.6	48.2 ± 12.4	47.4 ± 13.9	0.723
BMI (kg/m^2^)	20.1 ± 2.8	20.2 ± 2.6	20.1 ± 2.9	0.822
Smoking	13 (5.8)	6 (13.6)	7 (3.9)	0.013^*^
HbA1c (%)	9.0 ± 1.6	10.0 ± 2.1	8.8 ± 1.4	0.001^*^
HbA1c (mmol/mol)	77 ± 20	89 ± 28	74 ± 16	
C-peptide (nmol/l)	0.3 ± 0.2	0.3 ± 0.3	0.2 ± 0.2	0.058
**BLOOD PRESSURE**				
SBP (mmHg)	118.4 ± 15.1	124.6 ± 13.5	116.9 ± 15.1	0.001^*^
DBP (mmHg)	72.0 ± 11.3	79.0 ± 11.4	70.3 ± 10.6	0.001^*^
**LIPID PROFILE**				
Total cholesterol (mg/dL)	170.0 ± 29.9	180.2 ± 36.6	167.5 ± 27.5	0.034^*^
HDL-C (mg/dL)	65.7 ± 12.5	65.6 ± 13.4	65.7 ± 12.4	0.980
LDL-C (mg/dL)	97.6 ± 28.0	106.6 ± 34.9	95.4 ± 25.7	0.050
Triglyceride (mg/dL, log-transformed)	4.2 ± 0.4	4.4 ± 0.5	4.2 ± 0.3	0.003^*^
Non-HDL-C (mg/dL)	107.3 ± 29.8	120.4 ± 34.4	104.3 ± 27.9	0.007^*^
Total cholesterol to HDL-C ratio	2.66 ± 0.6	2.87 ± 0.75	2.61 ± 0.56	0.037^*^
Triglyceride to HDL-C ratio	1.21 ± 0.98	1.64 ± 1.41	1.10 ± 0.82	0.019^*^
Urine albumin to creatinine ratio (mg/g)	90.29 ± 459.4	424.85 ± 975.63	8.51 ± 5.18	0.007^*^
**INSULIN INJECTION (TIMES/DAY)**				
2 or less	109 (48.7)	29 (65.9)	80 (44.4)	0.011^*^
3 or more	115 (51.3)	15 (34.1)	100 (55.6)	
Insulin dose/weight (U/kg)	1.10 ± 0.23	1.17 ± 0.23	1.08 ± 0.22	0.031^*^
Diabetic retinopathy	34 (15.2)	15 (34.1)	19 (10.6)	<0.001^*^

*Data are expressed as the n (%) or mean ± SD or year (inter-quartile range, IQR). n = numbers of patients with information for each separate variable*.

* *p-value < 0.05. DN, Diabetic nephropathy*.

The proportion of female was higher in the patient with DN than without DN groups (61.4 vs. 40.0%, *p* = 0.011) (Table 1). A higher proportion of the patients with DN smoked cigarettes (13.6–3.9 % in patients with and without DN, respectively, *p* = 0.013). The patients with DN had poor metabolic control, as indicated by elevated HbA1c levels [10.0 ± 2.1 % (89 ± 28 mmol/mol) vs. 8.8 ± 1.4 % (74 ± 16 mmol/mol) for patients with and for those without DN, respectively, *p* = 0.001]. The patients with DN had higher BP (systolic and diastolic pressure, 124.6 ± 13.5 vs. 116.9 ± 15.1 mmHg, *p* = 0.001 and 79.0 ± 11.4 vs. 70.3 ± 10.6 mmHg, *p* = 0.001, respectively), TC levels (180.2 ± 36.6 vs. 167.5 ± 27.5 mg/dL, *p* = 0.034), TG (4.4 ± 0.5 vs. 4.2 ± 0.3, log-transformed, *p* = 0.003) and non-HDL-C levels (120.4 ± 34.4 vs. 104.3 ± 27.9 mg/dL, *p* = 0.007) than those without DN. Both the TC-to-HDL-C and TG-to-HDL-C ratios were higher in the patients with DN (2.87 ± 0.75 vs. 2.61 ± 0.56, *p* = 0.037 and 1.64 ± 1.41 vs. 1.10 ± 0.82, *p* = 0.019, respectively). Moreover, a higher number of the patients without DN received multiple insulin injections per day (≥3 times per day, *p* = 0.011), although these patients had lower insulin doses per weight (1.17 ± 0.23 and 1.08 ± 0.22 U/kg for patients with and for those without DN, respectively, *p* = 0.031). Additionally, the prevalence of DR was significantly higher in the patients with DN (34.1% and 10.6% in patients with and in those without DN, respectively, *p* < 0.001).

To assess the risk factors for DN, we performed a Cox proportional hazards model analysis (Table 2). The univariable analysis revealed that female, smoking, high HbA1c, BP, lipid levels, and insulin dose were associated with a higher risk of DN (*p* < 0.05). In contrast, multiple insulin injections per day (3 or more times per day vs. 2 or less times per day) provided protection against DN (HR 0.41, 95% CI 0.22–0.77, *p* = 0.005). In the multivariate analyses accounting for the aforementioned factors, female (HR 3.40, 95% CI 1.66–6.96, *p* = 0.001), smoking (HR 3.60, 95% CI 1.28–10.10, *p* = 0.015), HbA1c level (HR 1.27, 95% CI 1.07–1.49, *p* = 0.005), diastolic blood pressure (HR 1.06, 95% CI 1.03–1.09, *p* < 0.001), and insulin injection time per day (3 or more times per day vs. 2 or less times per day, HR 0.51, 95% CI 0.26–0.98, *p* = 0.043) remained significant factors.

**Table 2:** Cox proportional hazard ratios of risk factors for diabetic nephropathy.

	**Univariate analysis**			**Multivariate analysis**		
**Variable**	**Hazard ratio**	**95% CI**	***p***	**Hazard ratio**	**95% CI**	***p***
Sex (female vs. male)	2.14	1.17–3.95	0.014^*^	3.40	1.66–6.96	0.001^*^
Age at onset (≥ 5 vs. <5 years old)	1.25	0.57–2.71	0.580			
Smoking (yes vs. no)	3.56	1.50–8.46	0.004^*^	3.60	1.28–10.10	0.015^*^
HbA1c (%)	1.46	1.24–1.72	<0.001^*^	1.27	1.07–1.49	0.005^*^
**BLOOD PRESSURE**						
SBP (mmHg)	1.03	1.01–1.05	0.001^*^	1.01	0.98–1.03	0.583
DBP (mmHg)	1.07	1.05–1.10	<0.001^*^	1.06	1.03–1.09	<0.001^*^
**LIPID PROFILE**						
Total cholesterol (mg/dL)	1.01	1.00–1.02	0.009^*^	1.00	0.99–1.01	0.696
LDL-C (mg/dL)	1.01	1.00–1.03	0.004^*^	1.00	0.99–1.01	0.683
Triglyceride (mg/dL, log-transformed)	3.34	1.85–6.03	<0.001^*^	1.47	0.73–2.96	0.279
Insulin dose (U/kg)	1.02	1.00–1.04	0.046^*^	1.02	0.99–1.04	0.140
**INSULIN INJECTION (TIMES/DAY)**						
3 or more vs. 2 or less	0.41	0.22–0.77	0.005^*^	0.51	0.26–0.98	0.043^*^

*HDL, high-density lipoprotein; LDL, low-density lipoprotein; CI, confidence interval*.

*All data are shown as the mean ± SD or numbers with the percentages in parentheses*.

* *p-value < 0.05*.

**Figure 1:**
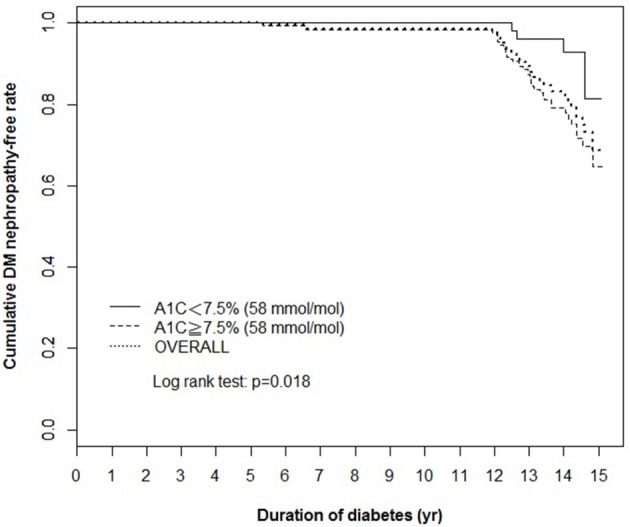
Kaplan–Meier cumulative survival curve of the total population (overall) and the population stratified according to the average HbA1c levels in last 5 years [<7.5% (58 mmol/mol) and ≥7.5% (58 mmol/mol)]. The cumulative survival rate of DN-free patients is plotted against the follow-up duration in years. The log-rank test was performed to compare the two groups. The dotted line represents the entire group, and the solid and dashed lines represent the two comparison groups in each figure. DN, diabetic nephropathy.

Using the clinically recommended HbA1c level 7.5% (58 mmol/mol) as cut-off point (2018), the Kaplan–Meier cumulative survival curve analysis showed a significant difference in the survival rates of the DN-free subjects at groups of the high and low average HbA1c levels in last 5 years (Figure 1).

## Discussion

The present study is the first to examine the risk factors for developing DN in patients with juvenile-onset T1DM in an Asian country. In the current study, 19.6% of patients with T1DM developed DN after disease duration of 12.6 years at mean onset of age 7.9 years. In the Oxford Regional Prospective Study, which was a large population-based cohort study in the United Kingdom that included 527 patients with T1DM with a mean onset of age 8.8 years, the cumulative prevalence of macroalbuminuria was 18% with a mean diabetes duration of 10 years (4). This prevalence was consistent with the prevalence reported in the current study.

Poor glycemic control is the most widely confirmed risk factor for DN in patients with T1DM. In the Oxford Regional Prospective Study, the cumulative risk of microalbuminuria increased by 36% for each 1% increase in the HbA1c level (18). Additionally, a study in Spain that enrolled 716 patients with T1DM revealed that an increment of 1% in the HbA1c level increased the risk of DN by 13% at 5 years and 68% at 10 years after onset (19). Our study supported this finding.

Additionally, smoking was a significant factor, with HRs up to three times higher than those obtained in the multivariable analysis. The importance of smoking cessation in the juvenile population with T1DM was highlighted in this study. In children and adolescents with T1DM, avoiding the onset of additional CVD risk factors is essential. Smoking increases the risk of albuminuria onset; therefore, smoking avoidance is necessary to prevent both microvascular and macrovascular complications (20). In the current study, smoking was an independent risk factor for DN. Discouraging smoking in young people who do not smoke and encouraging smoking cessation in those who do smoke are both crucial for the management of juvenile-onset T1DM. Smoking can induce a rise in systolic blood pressure and heart rate, while vascular leakage of albumin and glomerular filtration rate remain unchanged in normotensive T1D patients with microalbuminuria who had been smoking for years (21). The control of blood pressure was thus important for the prevention of diabetic nephropathy in T1D patients with smoking.

Many recent studies have reported a high prevalence of lipid abnormalities in adolescent populations with T1DM (22, 23). Data from the Oxford Regional Prospective Study revealed that 15.3% of patients had TC levels >5.2 mmol/L (200 mg/dL), and 17.9% of patients had TG levels >1.7 mmol/L (150 mg/dL). In the Nephropathy Family Study cohort, which analyzed 895 adolescents (aged 10–16 years) with T1DM, a significant proportion of patients exhibited sustained lipid abnormalities. In that study, the mean TC and non-HDL-C levels were higher in the patients with microalbuminuria than in those with normal albuminuria (22). In our study, the TC, non-HDL-C, and TG levels were all higher in the patients with DN than in those without DN. The difference in LDL-C level did not reach the statistical significance with the *p* < 0.05 in the current study. Although the ADA guidelines (2017) recommend pharmacotherapy for children with LDL-C levels >160 mg/dL or LDL-C levels >130 mg/dL and one or more cardiovascular disease (CVD) risk factors, the mean LDL-C level in the patients with DN was higher than the recommended goal of a LDL-C level <100 mg/dL for children with T1DM. Population-based studies have estimated that 14–45% of children with T1DM have two or more CVD risk factors (24, 25). Additional research is necessary to determine whether clinicians should treat dyslipidemia more stringently in juvenile-onset T1DM patients than the ADA recommendations.

The blood pressure was increased as the diabetic nephropathy was present due to the activation of renin-angiotensin aldosterone system (RAAS) and inflammation in glomerulus. Hypertension is a strong risk factor for the development and progression of diabetic kidney disease (26). The relation of elevated blood pressure with diabetes nephropathy is characterized by retaining of sodium concentrated fluids and as well as marginal vascular resistance (27). The current data was compatible with these reports.

In the Diabetes Control and Complications Trial (DCCT), 52% of patients with poor metabolic control had T1DM without complications. Conversely, 15% of patients with good metabolic control developed DN over a 9-year follow-up period (28). The type of treatment and glucose variation may also play crucial roles in the development of DN. Because considerable glucose fluctuations and varying basal insulin demands are observed across the developmental stages of juvenile-onset diabetes, the appropriate choice of treatment is critical for glucose control (29, 30). Recent advances in insulin analog use and the increased use of devices for self-monitoring of blood glucose could have contributed to the relatively low incidence of DN in our cohort compared with the DCCT cohort.

The DCCT and Epidemiology of Diabetes Interventions and Complications (EDIC) study (31) demonstrated the benefits of intensive treatment for T1DM. In our study, patients receiving multiple injections per day had an 49% lower risk of DN than those receiving the conventional number of injections per day (1 or 2 per day). This finding may be explained by more effective glycemic control in patients who receive multiple injections per day. Studies have demonstrated that these patients also experience fewer glycemic fluctuations, which are also a risk factor for microvascular complications in adolescents with T1DM (32, 33).

An older age at onset of T1DM (>5 years) was associated with an increased risk of DR in our previous study (13). However, the risk of DN was not significantly increased. Although the risks associated with different ages of onset of T1DM have been analyzed using various methods, the results have been inconclusive. Some studies have reported that the risk of DN is lower in patients with a T1DM onset before the age of 5 years (34). Other studies have shown that the overall cumulative risk is similar after a 10-years duration of diabetes despite early differences based on the age of onset (<5, 5–11, and >11 years) (18). In the current study, 81.8% of the patients with DN had an age of onset ≥5 years, whereas only 18.2% of the patients had an age of onset <5 years; however, this difference was not significant in the Cox regression analysis (HR 1.25, *p* = 0.580). Because DN primarily develops after puberty, the patients with an older age at T1DM onset were more likely receive a DN diagnosis because they were more likely to reach the post-pubertal stage at the end of follow-up. However, the difference was not sufficiently large in the current study.

In adolescents, previous studies suggested that female sex increased the risk of microalbuminuria in type 1 diabetes (18, 35). In our current study, the female has a higher risk of developing nephropathy with HR 2.14 in childhood-onset type 1 diabetes. The mechanism underlying the difference in the development of diabetic nephropathy related to female could be epigenetic modifications (36, 37). Hormonal changes for sexual maturation during puberty and high glucose exposure may induce epigenetic modifications differentially between two sexes. The occurrence of juvenile-onset type 1 diabetes could thus lead to different risk profiles between men and women. In addition, sex influence on nephropathy has been found to be age dependent and men are at higher risk to develop kidney complications in adult type 1 diabetes (38).

Regarding the association between DR and DN, the progression of one disease increased the risk of the other in the DCCT (39) regardless of the presence of established risk factors for microvascular complications. In our study, the incidence rates of DR in the patients without and with DN were 10.6 and 34.1%, respectively. The association of diabetic nephropathy and retinopathy was moderate in juvenile-onset type 1 diabetes. These findings suggest that the presence of either DR progression or DN development may indicate a need for closer monitoring for the incidence of other complications.

The strength of this study was that the identification of DN onset was robust because it was based on careful and regular examination under the DSCP. The sufficient follow-up period for medical records, laboratory data, and glycemic control in most of the patients enabled us to perform risk factor analysis based on data collected before the onset of DN. The patients were followed up at a fixed center that was inspected by the national health insurance infrastructure. Thus, the data included in the analysis were consistent, and any variability related to changes in methods over time or the data collection method was limited. The weaknesses of the study are the retrospective observational design and the relatively small sample size compared with other studies on adult Caucasian patients with T1DM. However, the incidence of juvenile-onset T1DM is lower in Asian populations than in Caucasian populations. It is the largest cohort study of diabetic nephropathy for juvenile-onset T1DM in Taiwan. We have no subjects with diabetic nephropathy proven by renal biopsy and their prevalent pathology. But albuminuria is the most accessible surrogate marker of diabetic nephropathy in such a cohort study of these juvenile-onset type 1 DM patients.

In conclusion, female, smoking, high HbA1c, diastolic blood pressure levels were associated with an increased risk of DN and intensive therapy was a protective factor for DN in Taiwanese children and adolescents with T1DM. This study is the first representative cohort study of the risk factors for DN in Asian patients with juvenile-onset T1DM in the post-DCCT era.

## Author contributions

C-CY and C-HL wrote manuscript, researched data equally, N-KW contributed to analysis and interpretation of data, C-CL contributed to the study conception and F-SL reviewed/edited manuscript. All authors were involved in the interpretation of data, critical revision, and approval of the manuscript. F-SL is the guarantor of this work and, as such, had full access to all the data in the study and takes responsibility for the integrity of the data and the accuracy of the data analysis.

### Conflict of interest statement

The authors declare that the research was conducted in the absence of any commercial or financial relationships that could be construed as a potential conflict of interest.
